# Automated machine learning for genome wide association studies

**DOI:** 10.1093/bioinformatics/btad545

**Published:** 2023-09-06

**Authors:** Kleanthi Lakiotaki, Zaharias Papadovasilakis, Vincenzo Lagani, Stefanos Fafalios, Paulos Charonyktakis, Michail Tsagris, Ioannis Tsamardinos

**Affiliations:** Department of Computer Science, University of Crete, Heraklion, Greece; Department of Computer Science, University of Crete, Heraklion, Greece; JADBio Gnosis DA S.A., Science and Technology Park of Crete, GR-70013 Heraklion, Greece; Laboratory of Immune Regulation and Tolerance, School of Medicine, University of Crete, Heraklion, Greece; Biological and Environmental Sciences and Engineering Division (BESE), King Abdullah University of Science and Technology KAUST, Thuwal 23952, Saudi Arabia; SDAIA-KAUST Center of Excellence in Data Science and Artificial Intelligence, Thuwal 23952, Saudi Arabia; Institute of Chemical Biology, Ilia State University, Tbilisi, Georgia; Department of Computer Science, University of Crete, Heraklion, Greece; JADBio Gnosis DA S.A., Science and Technology Park of Crete, GR-70013 Heraklion, Greece; JADBio Gnosis DA S.A., Science and Technology Park of Crete, GR-70013 Heraklion, Greece; Department of Computer Science, University of Crete, Heraklion, Greece; Department of Economics, University of Crete, Heraklion, Greece; Department of Computer Science, University of Crete, Heraklion, Greece; JADBio Gnosis DA S.A., Science and Technology Park of Crete, GR-70013 Heraklion, Greece

## Abstract

**Motivation:**

Genome-wide association studies (GWAS) present several computational and statistical challenges for their data analysis, including knowledge discovery, interpretability, and translation to clinical practice.

**Results:**

We develop, apply, and comparatively evaluate an automated machine learning (AutoML) approach, customized for genomic data that delivers reliable predictive and diagnostic models, the set of genetic variants that are important for predictions (called a biosignature), and an estimate of the out-of-sample predictive power. This AutoML approach discovers variants with higher predictive performance compared to standard GWAS methods, computes an individual risk prediction score, generalizes to new, unseen data, is shown to better differentiate causal variants from other highly correlated variants, and enhances knowledge discovery and interpretability by reporting multiple equivalent biosignatures.

**Availability and implementation:**

Code for this study is available at: https://github.com/mensxmachina/autoML-GWAS. JADBio offers a free version at: https://jadbio.com/sign-up/. SNP data can be downloaded from the EGA repository (https://ega-archive.org/). PRS data are found at: https://www.aicrowd.com/challenges/opensnp-height-prediction. Simulation data to study population structure can be found at: https://easygwas.ethz.ch/data/public/dataset/view/1/.

## 1 Introduction

Genome-wide association studies (GWAS) identify genetic loci associated with a specific trait or disease by scanning multiple markers across the genome. Although GWAS have led to the discovery of hundreds of thousands of risk variants (https://www.ebi.ac.uk/gwas/), the large numbers of false positives and the tiny effect size of most of these variants hinder their clinical use.

Translational genomics and precision medicine both promise to improve healthcare by bridging the gap between the lab and the clinic (Zeggini *et al.* 2019). Sporadic examples of success stories of GWAS for the clinic already exist for Mendelian diseases and pharmacogenomics (the identification of genetic variants that influence drug response) (Potamias *et al.* 2014) but for common complex diseases where multiple genetic and environmental factors affect disease risk, a systematic translation of research findings to clinical application demands not only large-scale genomic data linked to detailed electronic health records, but also robust statistical methods that will automate the entire data analysis life cycle.

The most studied genetic variants in GWAS are single-nucleotide polymorphisms (SNPs). Early GWAS were testing one SNP at a time using univariate regression. Today, most approaches are based on linear mixed models (LMMs) with random effects (Zhang *et al.* 2010, Loh *et al.* 2018, Runcie and Crawford 2019, Uffelmann *et al.* 2021). However, these methods are both computationally challenging for large datasets, and cannot distinguish causal SNPs from nearby variants, since neither are independent of the phenotype. Fine-mapping methods have been developed to identify causal SNPs in a post-hoc analysis (Schaid *et al.* 2018). However, as genomic data increase in sample size, linkage disequilibrium becomes more apparent and fine-mapping tools face an increasingly complicated task of refining wider regions.

For translational genomics, discovering causal variants is not enough. Computing the individual genetic liability for a given trait is also essential. For complex diseases, the cumulative small effect of many variants often defines the individual disease risk. Using GWAS data, polygenic risk score (PRS) analysis is performed that aggregates the effects of variants across the genome to predict phenotypes based on genetic profile (Dudbridge 2013).

In PRS computation, however, different protocols for performing analyses often lead to inconsistency between studies and misinterpretation of results (Choi *et al.* 2020). Reproducibility and replicability in GWAS has been a major challenge over the years (Huffman 2018) and is directly related to generalization in machine learning.

Machine learning (ML), the development and application of algorithms that learn from past data to make future predictions, have already been proved effective in the analysis of large, complex datasets, and is likely to become ever more important to genomic data (Libbrecht and Noble 2015, Ho *et al.* 2019). In ML, feature selection, which aims at identifying the most important features in a dataset, discarding those that are irrelevant or redundant (Guyon and Elisseeff 2003), is of specific interest for GWAS data which combine a high number of features (*p*) and a low sample-size (*n*), or as it is called “large p, small n” setting, in the field of statistical machine learning. This high-dimensional, low sample-size setting drastically limits the power of general purpose statistical and ML approaches, and since there is a broadening gap between the number of features we are able to measure for a given sample (easily reaching tens or hundreds of millions with current technologies) and the number of samples we can collect (more commonly in the order of hundreds or thousands, or even as low as a few dozens in the case of rare diseases), feature selection becomes a crucial step in genomic data analysis.

Additionally, the presence of correlation between features and samples, due to linkage disequilibrium and population structure, respectively, combined with the existence of joint effects, either linear or nonlinear, like epistasis, make the analysis of genomic data even more challenging. The complex, highly interrelated biological and environmental network among human traits is directly related to the multiple feature selection in ML, the discovery of non-redundant sets of features that are equally predictive of the trait under investigation, a.k.a. statistical signatures or biosignatures for biological data. In our prior work we show empirical evidence that multiple biosignatures are indeed prevalent in omics data (Lagani *et al.* 2017).

Incorporating ML in genomic data analysis presents further burden from the ML side; manual construction of models requires significant statistical and coding knowledge, experience with the choice of algorithms and their tunable hyper-parameters, the feature selection process, and the estimation of performance protocols; furthermore, ML is prone to methodological errors that could lead to overfitting and overestimation of performance. Most importantly, ML requires significant time and effort.

The most recent solution to alleviate these problems comes from automated machine learning (AutoML) (Guyon *et al.* 2015, Hutter *et al.* 2020), a quickly rising sub-field of machine learning that tries to address the theoretical and algorithmic challenges, as well as create systems, that fully automate the ML process end-to-end. AutoML improves the productivity of the model development process in a way that minimizes errors and biases. AutoML automates algorithm selection, hyper-parameter tuning, performance estimation, and result visualization and interpretation. In this way, AutoML tools promise to improve replicability of the statistical analysis, deliver reliable predictive and diagnostic models that can be interpretable to a non-expert, while drastically increasing the productivity of expert analysts.

We propose the use of an AutoML tool, named Just Add Data Bio (JADBio) (Tsamardinos *et al.* 2022) to analyze genomic data. JADBio has been validated by the machine learning and statistical community and has been successfully applied to biological and medical data, e.g. protein function prediction (Orfanoudaki *et al.* 2017), breast cancer prognosis and drug response prediction (Panagopoulou *et al.* 2019), tissue-specific methylation biosignature discovery (Karaglani *et al.* 2022a), predictive modeling for: early and late mortality for patients with thrombosis or cancer (Danilatou *et al.* 2022), early diagnosis of type 2 diabetes (Karaglani *et al.* 2022b), COVID-19 (Nagy *et al.* 2021, Papoutsoglou *et al.* 2021, Bowler *et al.* 2022), non-small cell lung cancer (Rounis *et al.* 2021), autism diagnosis (Batsakis *et al.* 2021), and also to other scientific fields such as nanomaterial property predictions (Borboudakis *et al.* 2017), suicide prediction (Adamou *et al.* 2019), speech classification, or bank failure prediction (Agrapetidou *et al.* 2021).

In this work, we customized JADBio to include a feature selection algorithm named εpilogi (standing for selection in Greek), a variant of γ-OMP (Tsagris *et al.* 2022) that returns multiple feature subsets that are equally predictive. While for prediction purposes all these subsets are equivalent, it is important to inform the user of their presence for knowledge discovery purposes. Specifically, two variants *A* and *B* may be informationally equivalent for the outcome; however, only one of them may be causal. Hence, it is important to report both of them as being equivalent. JADBio equipped with εpilogi (hereafter simply referred to as JADBio-Gεn) is optimized to apply to the low- sample, high-dimensional omics data and thus makes it an ideal choice for genome-wide data analysis. In addition to predictive and diagnostic models ready for clinical use, JADBio-Gεn also returns the corresponding multiple—statistically equivalent—biosignatures, a notion that is not currently considered in GWAS, although it could be proved extremely important due to genetic redundancy. JADBio-Gεn automatically selects the best model and generates unbiased estimates of the mean performance and 95% confidence intervals. Experiments on simulated data, as well as on real human data from the European Genome-Phenome Archive (EGA, https://ega-archive.org/) and OpenSNP (Greshake *et al.* 2014), prove that JADBio-Gεn creates predictive models of high predictive performance, discovers causal variants, selects parsimonious sets of variants, and is exclusively data-driven with no need of prior knowledge.

## 2 Materials and methods

### 2.1 AutoML as implemented by JADBio

JADBio is an AutoML platform that, given a dataset and a selected outcome, returns among others (i) the best-found ML or statistical predictive model for the outcome, (ii) a selected minimal-size feature subset that leads to the winning model, and (iii) out-of-sample (i.e. on new data) estimates of the performance of the model. A full presentation of JADBio is in Tsamardinos *et al.* (2022). JADBio may try hundreds or thousands of ML pipelines (called ‘configurations' in this context) on a given problem. Each configuration consists of a pipeline of algorithms for preprocessing, feature construction (for complex data types), imputation, feature selection, and modeling and a choice for the values of their hyper-parameters. An example of a configuration is “impute missing values with their mean, run the εpilogi algorithm for feature selection with hyper-parameter values ΔBIC = 2, and equivalence threshold = 0.05, then run a Support Vector Machine with linear kernel and cost hyper-parameter *C* = 100.” Typically, the number of possible configurations ranges between a few tens to a few thousands. The system automatically decides which algorithms to try and which hyper-parameter values. It also decides how to evaluate the performance of the models produced by that configuration using (repeated) K-fold cross-validation or a hold-out.

The winning configuration is applied on all available data to (i) produce the final model and (ii) select features in the feature selection step. Hence, JADBio does not “lose samples to estimation” as it uses all data for the training of the final model (Tsamardinos 2022). To estimate the performance of this final model JADBio employs the Bootstrap Bias Corrected Cross Validation (BBC-CV) estimate (Tsamardinos *et al.* 2018) that corrects cross-validation estimates for trying multiple configurations (called “winner’s curse” in statistics). JADBio has been shown not to over-estimate the predictive performance of the models produced in an extensive study with more than 360 omics datasets (Tsamardinos *et al.* 2022). Currently, for feature selection JADBio employs the εpilogi (details are presented below), Statistical Equivalent Signatures (SES, Lagani *et al.* 2017), and LASSO (Tibshirani 1996), for feature selection, and Decision Tree, Random Forest, Ridge Logistic Regression, Support Vector Machines with linear and nonlinear kernels, for modeling classification problems. For the experiments in this article, only the εpilogi algorithm for feature selection was employed, as it is the only one that scales to the sizes of GWAS. Also, we should denote here that, in this work, features correspond to genomic variants and we will be using these terms interchangeably.

### 2.2 Feature selection for GWAS and the *εpilogi* algorithm

GWAS measuring millions or even tens of millions of SNPs require highly scalable feature selection algorithms. In addition, when feature selection is employed for knowledge discovery and identifying important SNPs, one needs to solve the multiple feature selection problem and identify not a single optimal feature subset (signature), but ideally all feature subsets that lead to optimal predictions: it is misleading to return to the domain expert a set of SNPs as the only ones required for building an optimal predictive model, if there exist a second set of SNPs with equally good predictive power. εpilogi is such an algorithm developed as part of this study that scales to millions of features. In addition, it is an algorithm for solving the multiple feature selection problem. First, we describe how εpilogi identifies a single feature subset, and then how to extend it to identify multiple such subsets.

εpilogi is a greedy feature selection algorithm based on the generalization of the Orthogonal Matching Pursuit algorithm (Pati *et al.*1993) called the γ-OMP (Tsagris *et al.* 2022). γ-OMP generalizes the standard OMP to any type of outcome, any type of predictor feature, metric for measuring residuals, and predictive model used internally by the algorithm. The algorithm starts with an empty set of selected features. In each iteration it builds a predictive model (e.g. using logistic regression) with the selected features and computes the residuals of the model (e.g. deviance residuals or raw residuals) (Tsagris *et al.* 2022). Next, εpilogi selects as the next-best feature to include the one that is mostly correlated with the residuals. Intuitively, it selects the SNP that provides the most information about the errors of the current model, which should approximately be the SNP with the largest added value for the model. The algorithm terminates when a stopping criterion has been satisfied, namely the P-value testing whether the difference between the Bayesian Information Criterion (BIC) (Neath and Cavanaugh 2012) of the models with and without the next-best feature is significant at a given threshold. The threshold is automatically tuned by JADBio by trying various reasonable values. JADBio will calculate ΔBIC values that are directly affected by training sample size and P-values of an X^2^ distribution. Please refer to Supplementary Data for detailed information on the exact formula.

A major difference between εpilogi and γ-OMP is that the former has been extended to heuristically discover multiple equivalent feature subsets. Two SNPs *R* and *C* are ‘informationally equivalent' with respect to predicting a given outcome, when one can substitute the other in the set of selected SNPs, and still obtain a model that is statistically indistinguishable in terms of predictive performance. More details on the theory of multiple feature selection and informational equivalence is in Tsamardinos *et al.* (2017). The heuristic method to consider two SNPs *R* and *C* informationally equivalent given the current selected SNPs, ***S*** is determined as follows: first, the residuals *r* of the model using ***S*** are computed. Then, if the following two conditions hold *R* and *C* are considered equivalent: *Ind(R; r* *|C*) and *Ind(r; C* *|R*), where *Ind(R; r* *|C*) denotes the conditional independence of *R* with *r* given *C*. When linearity is assumed, the test can be implemented by testing for significance the corresponding partial correlation. The tests *Ind* return a P-value and independence is accepted when it is larger than a threshold. Intuitively, *R and C* are heuristically considered equivalent, if *C* is known, then *R* provides no additional information for the residuals *r*, and if *R* is known, then *C* provides no additional information for *r*. A similar technique has been employed in the Statistical Equivalent Signatures (SES) algorithm (Lagani *et al.* 2017). The pseudocode is provided in the Supplementary Data. The first signature returned by εpilogi without considering the feature equivalences is called the ‘reference signature'.

Regarding algorithmic complexity, εpilogi is independent on the number of samples and linearly dependent on the number of features. Moreover, εpilogi can be easily parallelized since in every iteration, the most correlated with the residuals variable enters the candidate set. This selection criterion allows splitting the datasets into separate chunks of features *C*, storing the most correlated variable in each chunk and selecting the one with the highest correlation coefficient across all chunks. The number of chunks, *C*, and the sequence of chunk processing is independent of the final selected variables, thus parallelization depends only on computing resources (e.g. a high-dimensional problem of 2 × 10^3^ samples and 10^6^ features, is solvable in a few minutes on a typical 16GB RAM personal computer).

### 2.3 JADBio-Gεn: AutoML for genetic data

We denote with JADBio-Gεn the version of JADBio equipped with only the εpilogi algorithm as the feature selection algorithm, and all other feature selection algorithms disabled. The set of predictive models to try and tune (e.g. Support Vector Machines, Random Forests, Ridge Linear Regression) remain the same as in the standard *JADBio*.

### 2.4 Multiple feature selection for detecting causal genetic variants

In GWAS, one seeks to detect the genetic variants that are causally related to the outcome. Feature selection has been theoretically connected to causality (Spirtes *et al.* 1993, Tsamardinos and Aliferis 2003) under some broad conditions (Tsamardinos and Aliferis 2003) and assuming there are no latent confounding factors, the data distribution can be represented by a Causal Bayesian Network, where the edges of the network denote direct causal relations. In this case, the minimal-size, optimally predictive feature subset (i.e. the solution of the feature selection problem) is the set of direct causes, direct effects, and the direct causes of the direct effects of the outcome, called the Markov Boundary (Pearl 2009). Since, no SNP can be causally affected by the outcome, in this domain, the Markov Boundary of the outcome consists only of its direct causes. If there are multiple Markov Boundaries, or the sample size is too small to statistically distinguish between the true Markov Boundary and some other feature subset (i.e. both subsets lead to models whose predictive power cannot be statistically distinguished) then the direct causes of the outcome are contained within the union of the Markov Boundaries. In cases of latent confounding factors being present (e.g. SNPs in linkage disequilibrium are correlated due to their proximity in the genome with distance being a confounding factor), then the selected feature subsets may contain confounded features that are not directly causally affecting the outcome. However, the causal variants are still guaranteed to be members of the union of the Markov Boundaries. In summary, under the standard assumptions and conditions of causal discovery and modeling (Spirtes *et al.* 1993, Lagani *et al.* 2016), an optimal multiple feature selection algorithm should select feature subsets that not only lead to optimal predictive models, but also, they contain the causal genetic variants.

### 2.5 Standard practices in selecting variants in GWAS

The standard practice in selecting variants in GWAS is not based on ML feature selection. First SNPs are filtered based on linkage disequilibrium, minimum allele frequency, and other factors to reduce their numbers (Uffelmann *et al.* 2021). This step may potentially lose useful information. Second, every variant’s association (correlation in a general sense) with the outcome is tested and a P-value is produced. These P-values stem from testing *pairwise associations* (a SNP with the phenotype). Hence, each SNP is considered in isolation and independently of any other variant. SNP-to-SNP correlations are ignored. Finally, a *P*-value threshold is determined (typically equaling 5 × 10^−8^) that controls for multiple hypothesis testing. All SNPs with *P*-values smaller than the threshold are accepted as correlated with the outcome and as potentially biologically important. We will call this practice standard GWAS selection.

The difference of standard GWAS selection against ML feature selection is that the later selects SNPs in a combinatorial, multi-variate fashion. This has two ramifications. First, only the SNPs that provide added value to the already selected SNPs and the corresponding predictive model are selected. Hence, an optimal feature selection algorithm not only removes informationally irrelevant SNPs, but also removes SNPs redundant for optimal prediction; in contrast, standard GWAS selection may include redundant SNPs. Second, SNPs with low association when examined in isolation (high *P*-value) may actually be highly predictive in combination with other SNPs. These SNPs will not be included by standard GWAS selection. For all these reasons, ML feature selection is expected to not only select fewer genetic variants, but also lead to more predictive models.

### 2.6 Polygenic risk score analysis

In contrast to variant selection in GWAS data, PRS does not aim to identify individual SNPs associated to a given phenotype, but aggregates information from SNPs across the genome in order to provide individual‐level scores of genetic risk.

To compute the PRS, one needs to first select variants based on the published literature and combine them using published effect sizes in a linear model. Therefore, PRS computation requires not only SNP values for all samples, but also a file containing the summary statistics for all SNPs, acquired from previous studies on a specific outcome, e.g. human height.

PRS is a single score value, for every sample, independently of the collection of samples. Depending on a *P*-value threshold, either a specific value, or a range of lower and upper values, PRS scores are based only on the SNPs that pass this filter (*P*-value information should be always available in summary statistic file). Therefore, PRS computation will exploit information from a potentially large list of SNPs, where the association with the outcome is derived univariately and requires an already well-studied phenotype.

Furthermore, methods that either control for linkage disequilibrium (LD), or shrink the effect size estimates are applied. Both methods are prone to parameter tuning, e.g. in the widely used C + T (clumping + thresholding) method [for details see (Choi *et al.* 2020)], the *P*-value threshold of variants to be included in the PRS score should be optimized. Additionally, those parameters may be incorrectly approximated when base and target samples are drawn from different populations or differ in size (Dudbridge 2013). In general, when hyper-parameter tuning is poorly performed, it may lead to overfitted, non-parsimonious predictive models, and to overestimation of their predictive performance (Tsamardinos *et al.* 2014). In our proposed AutoML approach, optimal hyper-parameter tuning is ensured without the need of advanced statistical or bioinformatics knowledge. Moreover, multi-variate variant selection by *εpilogi* is performed in a data-driven way without the need of extra files or prior knowledge.

## 3 Results

### 3.1 εpilogi discovers more predictive and causally related variants than QTCAT

In this section we compare εpilogi (Tsagris *et al.* 2022) with a state of the art method in discovering causal variants called Quantitative Trait Cluster Association Test (QTCAT) (Klasen *et al.* 2016). QTCAT is also a multi-variate feature selection algorithm, specifically designed for genetic variants and arguably, the algorithm mostly related to εpilogi. QTCAT accounts for population structure and has been shown to outperform linear mixed model approaches on simulated data, as demonstrated in (Klasen *et al.* 2016). Briefly, QTCAT works as follows: QTCAT starts by generating a hierarchical clustering of all covariates based on their correlations, followed by testing these clusters for significant associations to the response variable along this hierarchy. The lowest, still significant clusters in the hierarchy are the final result clusters, which include all those covariates that are significantly associated to the response variable.

To ensure a fair comparison we integrated both εpilogi and QTCAT in JADBio’s automated pipeline (for details see Supplementary Methods) and tested which method will select the most informative and the most causally related SNPs, after optimizing the modeling algorithm and its hyper-parameter values within JADBio. Notice that εpilogi returns multiple feature subsets that are informationally equivalent, but for a fair comparison with QTCAT we only use the first (reference) subset found by εpilogi*.*

To have a gold standard regarding which are the causal SNPs that the methods should identify, we applied the simulation procedure proposed in Klasen *et al.* (2016). Specifically, the simulator uses real SNP measurements, but simulates an outcome to be causally determined by a number of stochastically selected SNPs. The number of the causal SNPs is denoted as the “Number of SNPs” simulation parameter. The simulation parameter distribution, taking values gamma and Gaussian, determines how causal SNPs are selected depending on their position. The heritability h*^2^* parameter determines the explained variance of the outcome by the causal SNPs (h*^2^* = 1 implies the outcome is a deterministic function of the causal SNPs).

In the first set of experiments, we applied four different simulation scenarios (I–IV) as in Klasen *et al.* (2016), corresponding to different combinations of values of the simulation parameters. For each simulation scenario, we generated 50 simulated outcomes. We then compare the two methods in terms of their efficacy of discovering predictive sets of SNPs by computing the coefficient of determination *R^2^* of the best model produced by JADBio using the selected SNPs. Notice that an optimally predictive model can at most reach predictive performance *R*^2^ *=* *h*^2^ *=* 0.7. We also compare the methods in terms of their efficacy in identifying the causal SNPs, by computing the True Positive Rate (TPR) and False Discovery Rate (FDR) defined as the percentage of returned causal SNPs (true positives) out of all causal SNPs (positives) and the percentage of returned non-causal SNPs (False Positives) out of all SNPs returned, respectively.

**Figure 1.**
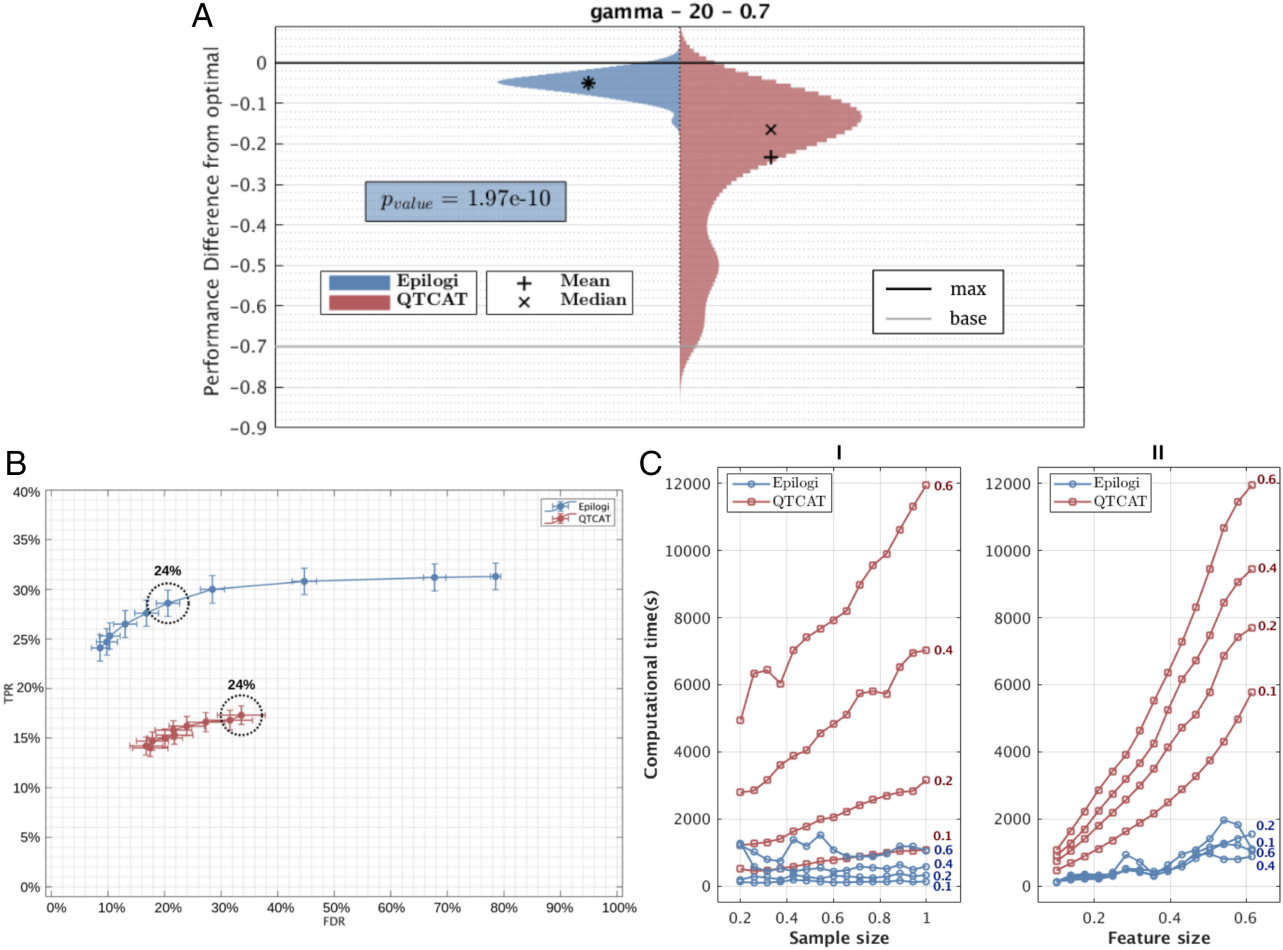
Comparison between εpilogi and GTCAT. (A) Distribution of differences of performances of the best models using signatures selected by εpilogi (left) and QTCAT (right) from the theoretical optimal model. The horizontal line base is the difference with the baseline model that always predicts the mean value of the outcome, and line max is the maximum difference from the optimal that can be achieved. The *P*-value of a *t*-test comparing the means of the distributions is shown. εpilogi discovers signatures that are statistically significantly more predictive than QTCAT. (B) Average True Positive Rate (TPR) and False Discovery Rate (FDR) of causal variants identification across 10 *P*-value thresholds for QTCAT and εpilogi. The threshold most frequently selected by JADBio when optimizing model performance is circled in dotted line, while the percentage of selection lies right above. εpilogi dominates QTCAT in both TPR and FDR. The threshold that most frequently optimizes performance achieves a balance between TPR and FDR, which is not true for QTCAT, while circle radius is inversely proportional to this frequency. (C) Computational time comparison between QTCAT and εpilogi. Left plot shows computational time for each feature selection method, as a function of relative sample size (100% corresponds to 1307 samples) for four different relative feature sizes (100% corresponds to 214 051 SNPs). The plot on the right shows computational time as a function of relative feature size for four different relative sample sizes. εpilogi scales better with both increasing sample size and feature size.

Figure 1A presents the predictive performance results for scenario I (Gaussian distribution for the SNP position, Number of SNPs: 20, and heritability *h*^2^: 0.7). The other scenarios produce qualitatively similar results and are shown in the Supplementary Data. Specifically, the y-axis corresponds to the difference of the predictive performance measured in *R*^2^ between the best JADBio model using the SNPs selected by εpilogi or *QTCAT* minus the best model using the truly causative SNPs. The *R^2^* performances are estimated on a hold-out test set of ∼130 samples. The distributions of performances over the 50 runs, along with their mean and median are presented for εpilogi and QTCAT, respectively. The figure contains two lines corresponding to 0 performance difference (achieved by the optimal model) and a baseline model, named max and base, respectively. The baseline model that achieves *R*^2^ = 0 (difference equals −0.7) is a model that always predicts the mean value of the outcome without consideration of any SNPs. The estimated performances above 0 (i.e. better than the theoretical optimum) are due to the estimation variance due to the finite size of the hold-out set. Estimates performances below 0.7 are due to either estimation variance or because the model is worse than the baseline model. The *P*-value of the paired *t*-test testing whether the average performance of models using SNPs selected by εpilogi equals the average performance of models using the *QTCAT* method is also reported in the figure (*P*-value = 1.97e−10) indicating the average performances are statistically significantly different. Importantly, εpilogi’s distribution of predictive performance, acquired from these 50 repeats, is of smaller variance, which is an indicative characteristic of a consistent model-producing methodology.

Figure 1B shows the trade-off between FDR and TPR achieved by the two algorithms for various *P*-value thresholds on the algorithms’ hyper-parameter values. We used 10 different values logarithmically spaced between 10^−6^ and 0.8 for both algorithms. Overall, εpilogi selects the causal variants with higher TPR and smaller FDR than *QTCAT* across all thresholds.

In a second set of experiments (see Fig. 1C), we compare the scalability in terms of sample size and feature space of *εpilogi* and *QTCAT*, for an arbitrarily chosen simulation scenario (distribution=gamma, number of SNPs=20, heritability=0.7). The maximum number of samples and features is limited by the size of the original SNP dataset to 1307 samples and 214 051 features. We vary the feature size within the range [10%, 60%] and sample size within [10%, 100%] or the original dataset and select features and samples with uniform probability. Each setting is repeated 50 times. As shown in Fig. 1C-left, εpilogi’s scales better with both increasing sample size and feature size. For the full dataset, *QTCAT* requires about 6 h of computational time. All simulations and experiments were performed on a machine with Intel Core i7-7700 processor running at 4.2 GHz with 32GB RAM, and 64 bit Windows 10.

We note that εpilogi and *QTCAT* are implemented in different programming languages (MATLAB and R respectively); hence, it is better to compare the slopes and scaling trends of the execution times, not the absolute times. In Fig. 1 we present the comparison between *εpilogi* and *QTCAT* in terms of (i) predictive performance, (ii) ability to detect true associations, and (iii) scalability in terms of computational time.

We applied four different simulation scenarios (I-IV). Results on all scenarios and details on the simulation parameters are described in Supplementary Data. In Fig. 1A, we show results of the Scenario I (Gaussian distribution for the SNP position, Number of SNPs: 20, and heritability *h*^2^: 0.7). Each simulation scenario was repeated 50 times. During each repeat we computed the relative performance (coefficient of determination *R*^2^) as the result of the regression between the ground-truth SNPs and the those resulting from each feature selection method. (We note that we use the value of the best configuration reported from the machine learning pipeline.) When plotting the distribution of these relative performances, we expect models that performed closer to the ground-truth model (i.e. the linear models built with ground-truth predictors) to lie near the zero value of y-axis (max line), while the worst ones to reside around $h^{2}$. The value of $h^{2}$ is the minimum theoretical performance (base) corresponding to the random guessing in ML terms. In practice, since $h^{2}$ is a statistical parameter, the minimum actual performance will vary around this parameter. Models that perform higher than the base line identify SNPs which are associated with the random independent noise dictated by $h^{2}$ parameter and this is a statistical artifact which should not be considered. Models with lower performance than the minimum actual performance select SNPs that systematically predict worse than using the average value of phenotype. (Negative values of $R^{2}$ can occur here. From the formula: $R^{2}=1-(\sum_{i}{\left(y_{i}-{\hat{y}}_{i}\right)}^{2})/(\sum_{i}{\left(y_{i}-\bar{y_{i}}\right)}^{2})$ this is apparent when $\left|y_{i}-{\hat{y}}_{i}\right|>\left|y_{i}-\bar{y}\right|$ is true on average.) *P*-value of the paired *t*-test, i.e. when testing the null hypothesis of equal performances between εpilogi and QTCAT method is also reported.

Regarding predictive performance, εpilogi’s reference signature produces models that are statistically significantly more accurate than the corresponding models of *QTCAT*, across all four simulation scenarios. More importantly, εpilogi’s distribution of predictive performance, acquired from these 50 repeats, is of smaller variance (distributions with tighter bounds), which is an indicative characteristic of a consistent model-producing methodology. With respect to ground-truth signature retrieval, εpilogi always detects more true positive features (higher TPR), in all selection thresholds. εpilogi also achieves lower FDR for the most qualified (most frequent hyper-parameter value used in best model) selection threshold.

To compare the efficiency of εpilogi and QTCAT on detecting true association, we calculated the true positive rate, (TPR) and false discovery rate, (FDR) (see Fig. 1B). We note that for *QTCAT* we used 10 *P*-values logarithmically spaced between 10^−6^ and 0.8 (we chose these boundary values to vary from a particularly strict, in terms of selection, scenario to including nearly all associations), while for *εpilogi* the equivalent ΔBIC scores range from 31 to 7.13, respectively (please refer to Supplementary Data for the detailed ΔBIC formula that generates these values directly from the *P*-value set).

In Fig. 1C, we examine the algorithmic (time) complexity for each feature selection method, i.e. the scalability in terms of sample size and feature space, by analyzing an arbitrarily chosen simulation scenario (distribution=gamma, number of SNPs=20, heritability=0.7). Since the simulation occurs only for the phenotype, the maximum sample and dimensionality size are initially limited to the corresponding size of the original dataset, that is 1307 samples and 214 051 features. Nonetheless, *QTCAT* requires a substantial amount of computational time to complete when feature size is maximum, approximately 6 h for 1307 × 214 051dataset. Thus, we constrained feature size to a range of 10%–60%, while sample size to a range of 10%–100%. Regarding time complexity, *εpilogi* is more efficient regardless of sample or feature space size. As shown in see Fig. 1C-left, *εpilogi’s* computational time is invariant of sample size, while QTCAT’s is linearly dependent, with increasing slope as sample size increases. Regarding feature size (see Fig. 1C-right) both methods have linearly dependent computational time; however again, the corresponding slopes are substantially larger for *QTCAT*. Since εpilogi and QTCAT are implemented in different programming languages in these experiments (MATLAB and R respectively), the absolute time differences between these methods should not be considered, rather than the differences between the respective derivatives (slopes), which capture the inherent Big-O notation of each algorithm.

### 3.2 εpilogi discovers more predictive and disease-related variants than standard GWAS variant selection

In this section, we compare the variants selected by εpilogi and standard GWAS selection with respect to the predictive power of the selections and their biological relevance. Same as in the previous experiment, for a fair comparison, both types of selecting variants are embedded within JADBio to optimize the final predictive model and estimate its performance. Specifically for standard GWAS selection, we directly selected the same variants as the ones reported in the published papers introducing the datasets employed for the comparison. On one hand, this direct selection ensures we apply the exact methodology of the authors of the published studies as intended. On the other hand, it is important to note that the variants selected in the publications are based on the same data that are being cross-validated during model optimization, i.e. variant selection is not cross-validated. Hence, the performance estimates reported for the standard GWAS selection are expected to be optimistic (Tsamardinos 2022) favoring this methodology.

The evaluation is performed on four real disease-related datasets, i.e. datasets where the outcome is the disease status, leading to binary classification task. The data has been deposited at the European Genome-Phenome Archive. EGA offers a vast amount of genotyped human samples diagnosed with a certain disease, alongside with control datasets such as 1958 British Birth cohort and National Blood Donors cohort. We analyzed datasets from the following human disease studies: (i) Ankylosing spondylitis-EGAS00000000104 (Evans *et al.* 2011), (ii) Multiple sclerosis**-**EGAS00000000101 (Sawcer *et al.* 2011), (iii) Parkinson’s-EGAS00000000034 (Spencer *et al.* 2011), and (iv) Psoriasis**-**EGAS00000000108 (Strange *et al.* 2010). In each study, a number of filters [e.g. Hardy–Weinberg equilibrium, minor allele frequency (MAF), etc.] that excludes either samples or variants, here SNPs, has been applied first, as indicated in the respective originally published study. This time, both the outcome and the variant data are real and not simulated; hence, the optimal predictive performance and the true causal variants are unknown.

To find the optimal model, JADBio trains tens of thousands of model instances produced by thousands of configurations (ML pipelines). As an example, in the psoriasis dataset, the analysis completed within 10 h after cross-validating 4340 configurations, producing 43 400 corresponding model instances. All runs took place on a machine running the Fedora OS with AMD Ryzen Threadripper 3960X 24-Core processor, and 128 Gb RAM. The comparison results are shown in Table 1. The results clearly demonstrate that *εpilogi* discovers variants that lead to more predictive models than the standard methodology outperforming standard GWAS selection by more than 25 AUC points in the Ankylosing Spondylitis dataset.

**Table 1.** Comparing predictive performance in four disease datasets.^a^

Disease	SNPs in reference signature (# of informationally equivalent SNPs)	Predictive performance—AUC [CI_AUC_]	Optimal classification model type
Variant selection by *εpilogi*			
Ankylosing spondylitis	13 (2)	*0.887 [0.861–0.914]*	Support Vector Machines
Multiple sclerosis	92 (19)	*0.823 [0.797–0.851]*	Random Forests
Parkinson’s	11 (8)	*0.758 [0.728–0.79]*	Ridge Regression
Psoriasis	27 (29)	*0.893 [0.865–0.907]*	Random Forests
Variant selection by standard GWAS practices as in the original published studies			
Ankylosing spondylitis	8	0.612 [0.574–0.649]	Random Forests
Multiple sclerosis	34	0.586 [0.546–0.625]	Random Forests
Parkinson’s	9	0.566 [0.527–0.605]	Random Forests
Psoriasis	17	0.752 [0.721–0.782]	Random Forests

a AUC refers to the Area Under the ROC Curve (higher is better, 1.0 is optimal) as our metric of predictive performance of accuracy. CI_AUC_ provides the 95% confidence interval of the predictive performance. Winning performances are designated with italics. JADBio-Gεn discovers more predictive sets of SNPs associated to the disease compared to the published studies. It also discovers SNPs that are informationally equivalent (i.e. one can substitute the other in the model and still obtain optimal performance) reported in the parenthesis.

There is no significant overlap between the SNP’s discovered in the original studies and those reported by εpilogi*.* This is explained by the fact that εpilogi removes redundant SNPs, as well as potentially including low association variants with high added value, as described above. However, we note that in the case of multiple sclerosis even though there are no common SNPs discovered initially, we did find five common genes selected after mapping those SNPs to their corresponding genomic regions.

To study disease association of the discovered SNPs, we measured the gene overlap in known pathways related to the disease studied. We consider related pathways as those including the disease term in their description. For example, for the ankylosing spondylitis disease we consider the following pathways: Ankylosing spondylitis in the Jensen_DISEASES library, Self-reported ankylosing spondylitis 20002 1313 and ICD10 ankylosing spondylitis M45 in the UK_Biobank_GWAS_v1 library, Ankylosing spondylitis in the PheWeb_2019 library, Ankylosing spondylitis in the DisGeNET library, Spondylitis, Ankylosing in the dbGAP, etc. We downloaded 193 different libraries including 382 983 pathways from the Enrichr database (Chen *et al.* 2013, Kuleshov *et al.* 2016, Xie *et al.* 2021). In Enrichr, each gene set is associated with a functional term or an enrichment term such as a pathway, cell line, or disease. We refer to any of those terms as entities. For each entity we counted how many genes that are linked to the discovered variants are involved in the entity.

**Figure 2.**
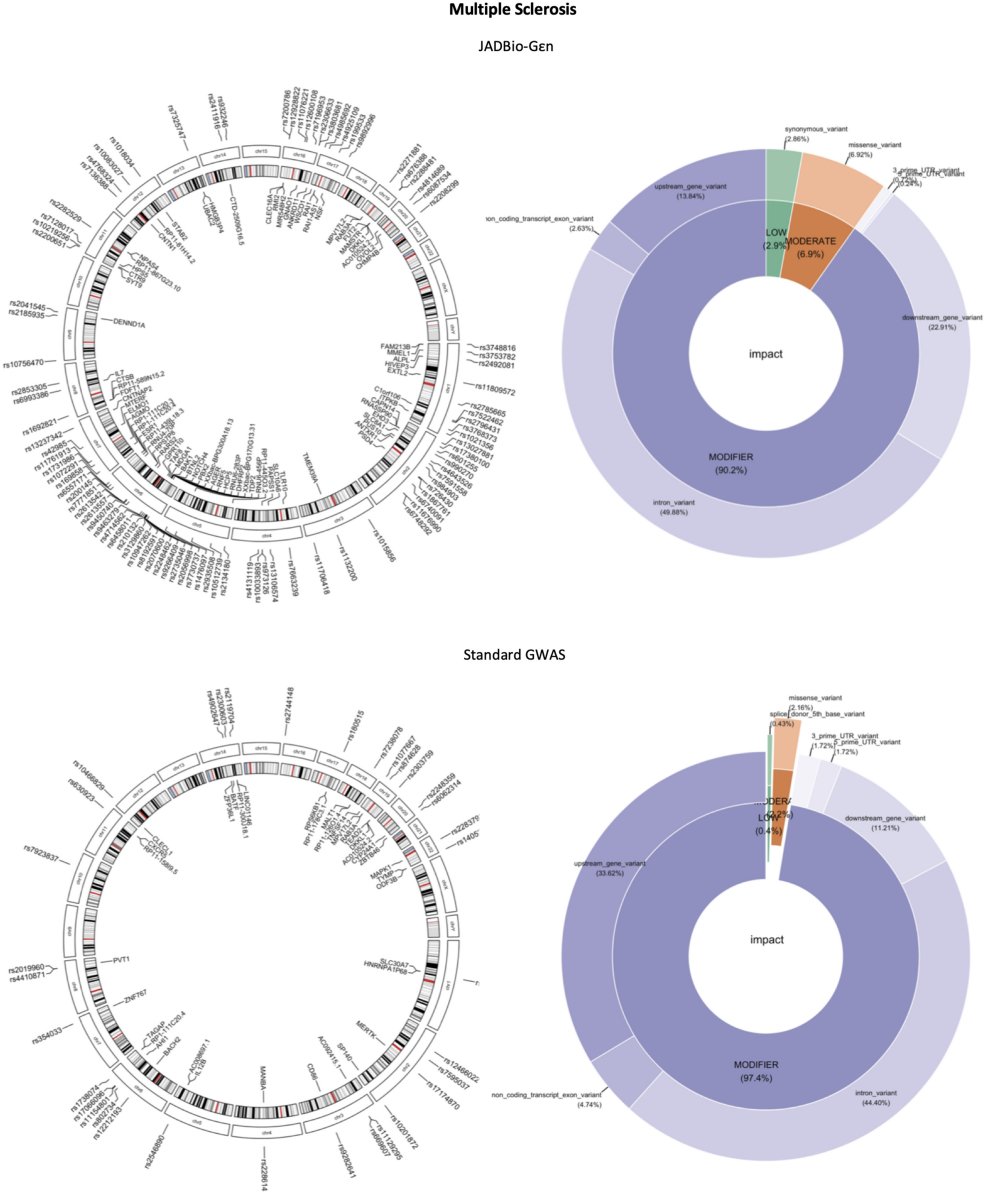
Genomic view of the variants and genes associated with multiple sclerosis (left) and their impact on protein function (right). Top left: Variants and genes discovered by JADBio- Gεn. Many variants lie in chromosome 6, the majority of which lies in the MHC which was the first susceptibility locus related to multiple sclerosis. Bottom left: Variants and genes discovered by the published study. JADBio-Gεn discovers more low or moderate impact SNPs than the original study and also a higher percentage of missense (6.9% versus 2.16%) variants (top and bottom right).

As shown in Table 2, εpilogi discovers more “disease-related” SNPs since they are found in more entities that have been related to the disease. To compare the biological impact of the discovered SNPs, we used Ensembl Variant Effect Predictor (VEP) and the Genome Reference Consortium Human Build 37 (McLaren *et al.* 2016), to determine any affected genes and the consequences of the variants on the protein sequence. Since many genes have more than one transcript, VEP provides a prediction for each transcript that a variant may overlap. We run VEP with the default settings, without filtering for consequence data per variant or gene transcription to allow for the maximum biological discovery. To visualize our data, we used circlize (Gu *et al.* 2014), biomaRt (Durinck *et al.* 2009), ggplot2 (Durinck *et al.* 2009) R packages, and a customized version of the PieDonut function in Gu *et al.* (2014). We also used the h19 cytoband data when needed. In Fig. 2, we provide circular genomic plots with cytoband data on the left to show where on the genome the discovered SNPs lie and pie and donut plots on the right to show the impact of those SNPs on protein function. Here, we show results for the multiple sclerosis (MS) disease. Similar plots for all other diseases can be found in Supplementary Methods. Most SNPs found in both cases are “Modifiers,” meaning that are usually non-coding variants or variants affecting non-coding genes, where predictions are difficult or there is no evidence of impact, according to VEP definitions. It is already known that most of the variants discovered by standard GWAS selection lie in non-coding regions making their functional interpretation challenging (Gu *et al.* 2014). With εpilogi, we discovered more missense variants compared to the original study. A missense variant is a sequence variant, that changes one or more bases, resulting in a different amino acid sequence but where the length is preserved, according to VEP definition. Also, many variants discovered by εpilogi lie in chromosome 6, the majority of which lies in the major histocompatibility complex (MHC) which was the first susceptibility locus related to multiple sclerosis (Patsopoulos *et al.* 2013).

**Table 2.** JADBio-Gεn discovers SNPs associated to genes that are involved in more related entities.^a^

Disease	Variants	Common variants	Genes	Common genes	Related entities in Enrichr	Related entities that include associated genes	Common entities
Variant selection by *εpilogi*							
Ankylosing spondylitis	**13**	0	**21**	0	13	**8**	3
Multiple sclerosis	**92**	0	**79**	5	65	**33**	13
Parkinson’s	**11**	1	**12**	2	139	64	62
Psoriasis	**27**	1	**21**	1	149	**49**	19
Variant selection by standard GWAS practices as in the original published studies							
Ankylosing spondylitis	8	0	8	0	13	5	3
Multiple sclerosis	34	0	37	5	65	25	13
Parkinson’s	9	1	11	2	139	**65**	62
Psoriasis	17	1	17	1	149	30	19

a Related entities are considered those that include the disease terms in their description. Bold indicates higher values when comparing JADBio results to those of the originally published studies.

### 3.3 Automated machine learning with JADBio-Gεn generalizes polygenic risk score analysis

In this section, we focus on the complete AutoML system, JADBio-Gεn, that includes not only the variant selection part (i.e. the εpilogi algorithm), but also the optimization of the hyper-parameters and the modeling step, as well as the estimation of predictive performance. We show that JADBio-Gεn generalizes the PRS analysis.

PRS analysis computes a multi-variate risk score for each new sample. This score could be used to directly classify new samples, but it is often employed as an extra predictive feature along the measured genetic variants.

Hence, as already mentioned, PRS computation requires prior knowledge of the variants associated with the outcome. Similarly, JADBio-Gεn computes a risk score using ML modeling and feature selection, but (i) it does not require prior knowledge about the variants to include in the final model; these are discovered by the feature selection algorithm using the data. (ii) It does not require knowledge of the coefficients of the selected variables; they are estimated from the data. (iii) It does not limit itself to a linear predictive model, but it explores several nonlinear ML models, such as Random Forests and nonlinear Support Vector Machines.

For the purposes of this section, we used data from the open online challenge on the CrowdAI platform (crowdai.org) aiming at predicting the height of an individual from genome-wide genotyping data. The initial dataset contained 7 252 636 variants which passed a quality threshold, defined as an imputation score INFO > 0.8, genotyping missingness frequency *F*_m_ < 0.1, and a Hardy–Weinberg equilibrium exact test P-value <10^−5^. Each genetic variant was represented by 0 (homozygous for reference), 1 (heterozygous), 2 (homozygous for the alternative allele), or NA (missing data or variants of allosomes). The data were partitioned by the challenge organizers into two sets, a training set with 784 samples and a test set of 137 samples.

The winning method in the competition was based on PRS using publicly available summary statistics of the GIANT study to achieve the best result (Naret *et al.* 2020). The training set and testing set were combined for quality control, data preparation, and gender imputation. Several preprocessing steps took place before modeling: (i) removing duplicate SNPs and invalid SNPs (i.e those without ids), (ii) keeping only SNPs that are common in train and test set, (iii) removing SNPs with multiple positions, and (iv) LD pruning (removing SNPs based on high levels of pairwise LD). LD pruning significantly shrinks feature size, from 6 854 199 variants that had left after the first three steps to 729 726. The winning model in the competition was a simple linear model including gender, three first principal components (PCs), and the PRS.

We reproduced all the steps of the winning method as stated in (Naret *et al.* 2020). These include: (i) gender imputation using PLINK (Dudbridge 2013), (ii) removal of related individuals using PLINK by computing identity-by-descent (IBD), which is a degree of recent shared ancestry. This analysis removed 24 individuals. The winner provided the indices of the individuals removed, therefore we removed the same individuals without reproducing this step, (iii) principal component analysis with PLINK, keeping the first three principal components to include in the model as proposed in the winning method, and (iv) PRS computation using PRSice (Dudbridge 2013). The winner computed a PRS using the training data at different P-value thresholds and then fitted a linear model on the training samples to select the P-value threshold with the highest additional variance explained. Then, using this *P*-value threshold they fit a linear model with all the covariates (gender, PCs, and PRS) to produce the final *R*^2^ = 0.53 on the test set.

To study the effects of the five covariates (gender, 3PCs, and PRS) we run JADBio-Gεn on the training data. We computed the PRS for 12 different *P*-value thresholds ranging from 10^−16^ to 1. The number of SNPs included in the PRS computation ranges from 98 to 91 260, respectively. We then run JADBio-Gεn on each dataset including all the five covariates. We found the best *R*^2^=0.495 on the test set when we used P-value threshold of 10^−10^ for the PRS computation including 230 SNPs. It is important to note here that JADBio-Gεn computes *R*^2^ exclusively on the test set to avoid any possible sources of overestimation of performance.

We also applied JADBio-Gεn on the initial dataset of 6 854 199 variants, without the preprocessing steps that significantly shrink the feature set. The question here is whether we can develop prediction models from SNP data only, without including any other covariates or prior knowledge. This run took 100 CPU hours. To reduce computational time in analyzing the entire dataset of 6 854 199 we set a threshold of maximum 50 SNPs to be selected. This run took place on a machine running the Fedora OS with AMD Ryzen Threadripper 3960X 24-Core processor, and 128 Gb RAM.

We achieved an *R*^2^ = 0.45 on the test data by using only SNP data. Although prediction accuracy is higher when using gender, PCs and the PRS, there are several disadvantages in including these covariates in the statistical analysis. First, the gender has been imputed in the dataset, increasing thus the stochasticity of the data, and the PCs have been computed on the entire dataset violating the golden rule in ML, that the test data cannot influence training the model in any way, introducing thus a source of bias. Last, PRS demands the existence of a well-studied phenotype and is not applicable in the absence of summary statistics, for example in the case of some rare or understudied phenotypes.

**Figure 3.**
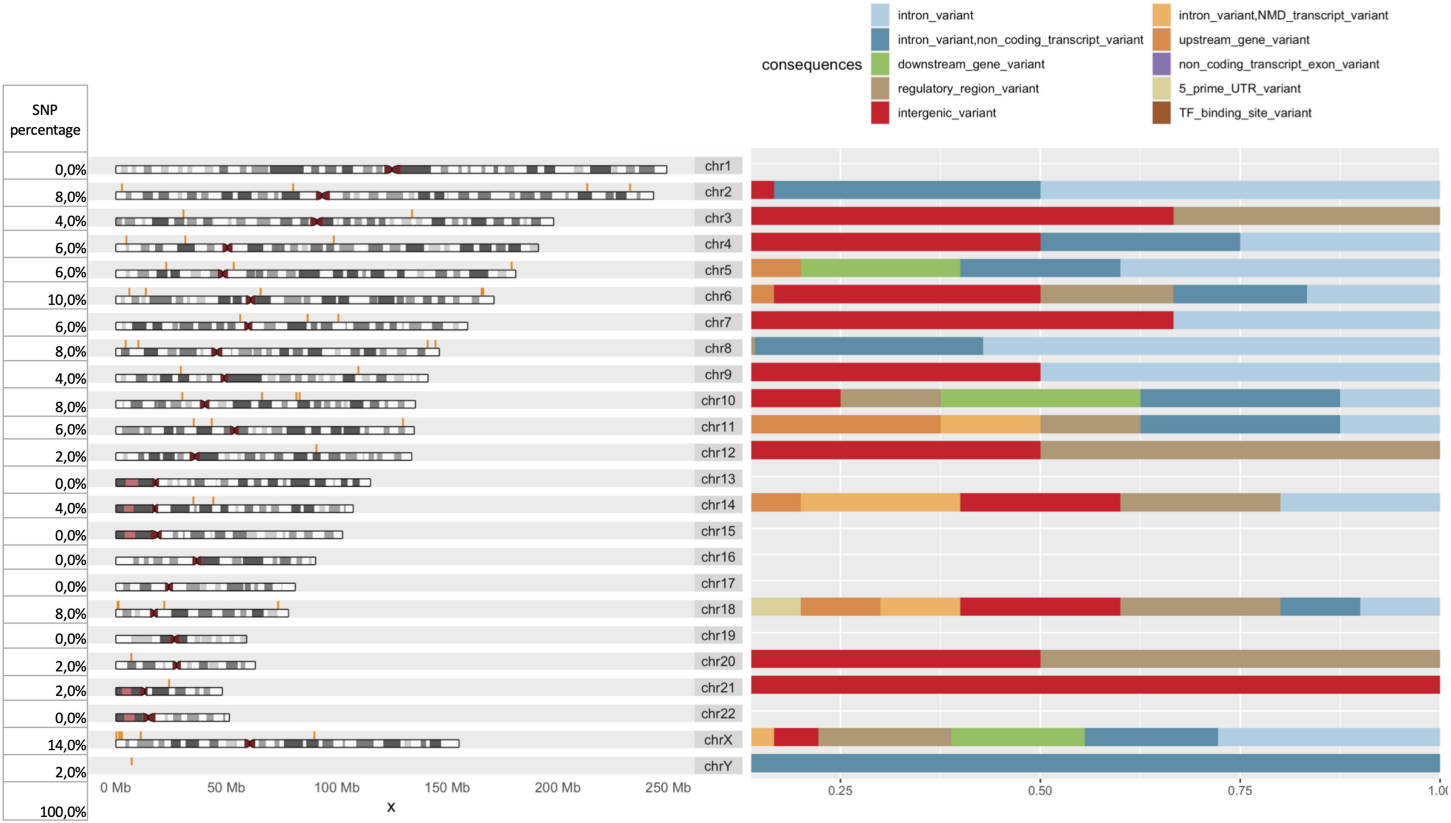
Chromosomal distribution and consequences of variants associated with height as detected by JADBio-Gεn. The left column shows the percentage of SNPs found to be associated with height in each chromosome. The most representative consequences of height variants include intronic variants (42%—light blue) or variants that are located in intergenic regions (32%—red), between genes.

In this work we also provide visual insights of the loci of the variants detected, accompanied by some functional annotation (i.e. variant consequences). In Fig. 3, we show the chromosomal distribution and consequences of variants associated with height as detected by JADBio-Gεn. Using VEP and the R visualization packages mentioned in the previous section we found all height variants have “modifier impact” and most of SNPs are found on the X chromosome.

## 4 Discussion

In this work, we compare εpilogi, a novel proposed multiple feature selection method included against QTCAT, an alternative GWAS multi-marker method proposed in the literature, in their ability to discover truly causal variants by comparing their TPR and FDR scores. εpilogi selected systematically more truly associated SNPs than QTCAT, while keeping FDR reasonably low. The TPR and FDR values improve further when considering the corresponding statistically equivalent features, pointing out again the importance of multiple signatures.

It is not trivial to identify molecular signatures in genomic datasets since multiplicity, a phenomenon where different analysis methods applied on the same or similar data, lead to different but apparently maximally predictive signatures, makes consistent generation of biological hypothesis very hard, hindering their translation to clinical practice (Statnikov and Aliferis 2010). This problem is particularly apparent in biology, where redundancy plays a key role to shield organisms against adverse events [see for example genetic redundancy (Nowak *et al.* 1997)]. Discovering multiple and statistically equivalent feature subsets has several advantages. Apart from increasing biological knowledge discovery, it may be proved very useful in biotechnology and translational genomics by offering different alternatives in designing measurement or diagnostic assays, or even drug targets, by considering different cost/effort solutions.

The εpilogi algorithm discovered multiple statistically equivalent solutions in all analyzed datasets. Specifically, in public disease datasets from EGA, we showed that those multiple alternative signatures map to similar biological entities. This indicates that there are many different genetic paths that may lead to the same phenotype. Methods that are able to discover most of those alternate genetic signatures provide valuable knowledge to life scientists and researchers.

Predictive performance is also important when it comes to computing individualized disease risk. We embedded εpilogi within an AutoML platform that we call JADBio-Gεn, that automatically optimizes the ML pipeline. Variants discovered with JADBio-Gεn led to predictive models of higher predictive performance than those discovered by standard GWAS in several disease studies. Furthermore, variants discovered by εpilogi, outperformed the ones discovered by QTCAT, in terms of predictive performance, in all simulation scenarios, in a consistent way.

In terms of time complexity, εpilogi proved to be far more computationally efficient than QTCAT, owing much of its superiority to the residual-based selection strategy. Arguably, QTCAT allocates high computational load to its initial hierarchical clustering step carried over a large portion of available SNPs. This clustering step is needed in order to deal with the multicollinearity present in the data. In contrast, εpilogi identifies correlated SNPs only for the few features that are included in the reference signature, forgoing unnecessary operations on unrelated SNPs. Furthermore, QTCAT uses an internal 10-fold cross-validation in order to tune the *λ* regularization parameter for LASSO selection procedure, thus burdened by additional operations on lower sample size parts of the data and although one may argue that QTCAT’s initial clustering is carried out only once for a given genomic dataset, in order to decrease computational time, this leads to a methodologically incorrect and biased data analysis, since cross-validation is performed on a prefiltering step on all available samples leading to information leakage on the subsequent analysis. Moreover, εpilogi can be easily parallelized by running the work separated into several chunks that proceed totally independently of one another, as described in Section 2.

Experiments on real, publicly available datasets showed that JADBio-Gεn discovers signatures of SNPs with systematically higher predictive performance than those reported in the standard GWAS. This means that there is hidden information in genomic data waiting to be discovered and that the most predictive signature is not always composed of the most associated SNPs, but rather by SNPs who complement each other in terms of informational content. It is actually possible that SNPs with no pairwise association with the phenotype to be necessary for optimal prediction, when considered jointly. JADBio-Gεn is able to recognize and filter out the redundant features and can be proved extremely useful also in combining genetic and clinical prediction models.

JADBio-Gεn can be efficiently used to provide genetic liability to a trait at the individual level. A major issue in PRS, is that it is computed based on summary statistics reported in the literature. Therefore, prior knowledge is necessary for PRS to work effectively. Another issue is that since the training is performed on the target dataset and the base data are only used to prefilter some SNPs, a third dataset is required to avoid overfitting. Another solution would be to split the target data and keep a separate hold-out set to estimate performance. These samples however are “lost to estimation” which is unacceptable in biomedical applications where sample collection is extremely difficult and costly. In rare diseases, for example, this would be almost impossible. JADBio-Gεn does not lose samples to estimation as it uses all data for the training of the final model, estimates out of sample predictive performance using advanced techniques, and does not require an external dataset for statistical validation of performance (Tsamardinos 2022). Specifically, it employs the BBC-CV estimate (Tsamardinos *et al.* 2018) to estimate the performance of this final model that corrects cross-validation estimates for trying multiple configurations. Also, JADBio-Gεn is an entirely data driven approach and does not need any prior knowledge to work.

## Supplementary Material

btad545_Supplementary_Data
