# Supplementary material for: Automated machine learning for genome wide association studies
Source: Bioinformatics. 2023 Sep 6;39(9):btad545. doi: 10.1093/bioinformatics/btad545 (PMC10562960; doi:10.1093/bioinformatics/btad545)
Supplement: btad545_Supplementary_Data [file btad545_supplementary_data.pdf]

Supplementary material

Description of public datasets from EGA

cases controls

**Disease**	**Samples**	**SNPs**	**Disease distribution**
Psoriasis	7.353	535.475	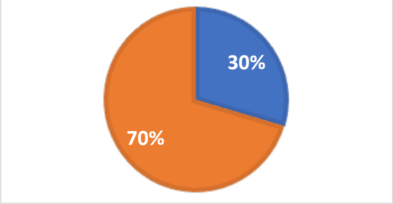
Parkinson's	6.880	532.588	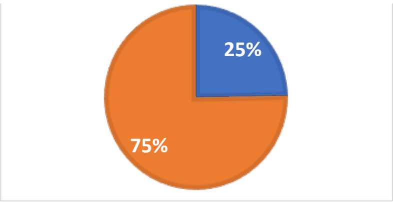
Ankylosing Spondylitis	6.588	487.780	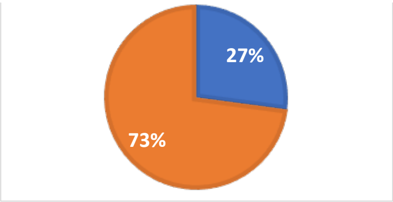
Multiple Sclerosis	15.474	475.806	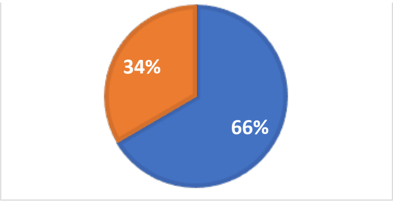

Pseudocode of the εpilogi algorithm

Algorithm 1 Epilogi

```
Input: X,   // a given dataset
Input: y,   // target
Input: stoppingCrit,  // a function which indicates if the FS process
                        should stop
remainingVars ← variables of X
equivalentVars← variables of X,  // initialize the set of equivalent
                                   variables for each variable
selectedVars← {},       // empty set of variables
curModel← y ~ selectedVars,  // fit null model

// Get residuals of current model
curResids← estimateResiduals(curModel, y)

// Find a model using the most correlated variable on the current
residuals
curVar← mostCorrelated(remainingVars, curResids)

// Fit a model using the selected variables and the current one
curModel← y ~ (selectedVars + curVar)

while (! stoppingCrit(curModel).holds) & len(remainingVars) > 0 do
        // Add curVar to selectedVars
        selectedVars← selectedVars + curVar

        // Remove curVar from remainingVars
        remainingVars← {remainingVars \ curVar}

        // Find equivalent variables to curVar
        equivalentVars← identifyEquivs(curVar,          remainingVars,
        curResids)

        // Remove equivalentVars from remainingVars
        remainingVars← {remainingVars \ equivalentVars[curVar]}

        curResids← estimateResiduals(curModel, y)

        curVar← mostCorrelated(remainingVars, curResiduals)

        curModel← y ~ (selectedVars + curVar)

Output: selectedVars, equivalentVars
```

Producing Multiple Signatures with *εpilogi*

We extended *εpilogi* to produce multiple signatures, namely multiple, predictive feature subsets whose performances are statistically equivalent, by iteratively identifying equivalent features to already selected ones as follows:

Let x be the selected variable and r_i the residuals of the fitted model. Variable y is considered equivalent to variable x, if both partial correlations corr(r_i, x|y) and corr(r_i, y|x) are not statistically significant.

Algorithm 2 identifyEquivs

```
   Input: curVar
   Input: remainingVars
   Input: curResids
   Input: equivThreshold,  // p-value

   Q← {},  // equivalent variables to curVar

   foreach (remainingVar ; R) do
        // Correlation of residuals and each remainingVar given curVar
               P1← corr(curResids, R | curVar),  // p-value

        // Correlation of residuals and curVar given each remainingVar
        P2← corr(curResids, curVar | R),  // p-value

        if (P1 > equivThreshold) & (P2 > equivThreshold)
              Q← Q + R

Output: Q
```

Simulation of structured populations

The simulation procedure which generates the phenotype is identical to (Klasen, J. R., Barbez, E., Meier, L., et al. (2016), Nat. Commun., 7, 1–8). The dataset used is acquired from the easyGWAS platform, available here and it consists of 1,307 genotyped samples of the species *Arabidopsis thaliana*. The simulation strategy exploits the real genetic profile of the samples in order to account for the underlying complicated mechanisms, such as heritability, in sets of populations. Alternatively, one could simulate the genotype as well, but that would require using models of mutation rate, crossover, etc., or even defining gene-rich regions, in order to produce realistic genetic profiles. Using real, genotyped data, overcomes this barrier and is only limited by the maximum number of samples used, in this case 1,307 which is statistically adequate.

Below we provide some basic information on this dataset.

Species	Arabidopsis thaliana

Dataset name	AtPolyDB (call method 75, Horton et al.)
Dataset build	TAIR9
#Samples	1,307
#Chromosomes	5
#SNPs	214,051
#SNPs in gene-rich regions	28,496
Dataset homozygous	YES

Firstly, a SNP "pool" is created from these SNPs that belong to previously known gene regions. This is to ensure that selected SNPs originate from areas that could affect the phenotype in a biologically realistic way. Next, probabilities dictated by a statistical distribution (Gaussian (normal), or gamma[1]), are assigned on each SNP position. Given a probability distribution, a predefined number of SNPs is drawn randomly acting as independent variables affecting the phenotype (dependent variable). The linear model's coefficient for each associative selected SNP is chosen at random from a Gaussian distribution. Finally, in order to avoid producing a deterministic phenotype relationship, a statistical noise parameter is added which simulates a random environmental effect and reflects the heritability of a set of SNPs. The magnitude of the noise parameter is such that it tends to match the unexplained variance of the linear model. In other words, the coefficient of determination, R^2 of this linear model, approaches the user-defined parameter, h^2. The simulated phenotype is continuous, resulting in a regression problem. Here we provide a flowchart of the simulation strategy.

[1] If gamma pdf is used, neighboring SNPs of a specific region have higher probability to be chosen as associative.

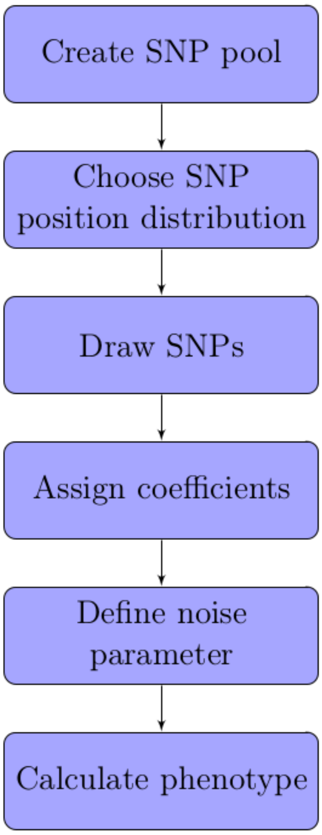

Comparison protocol for *QTCAT* and *εpilogi*

In order to provide a fair comparison between *εpilogi* and *QTCAT* (as feature selection methods), a common pipeline is set up. This is performed in terms of 1) parameters in simulation scenarios, 2) splitting of samples in train-validation-test sets, as well as in 3) modelling algorithms and their hyper-parameters. Regarding feature selection hyper-parameters, *εpilogi* and *QTCAT* require only one; 4) a selection threshold.

1. **Simulation Scenarios.** For phenotype simulation, 3 parameters are required; SNP position distribution (Gaussian or gamma), Number of associated SNPs and heritability, h^2 parameter. For the purposes of this evaluation, we concluded on 4 scenarios based on the aforementioned parameters;

    Distribution: Gaussian, Number of SNPs: 20, h^2: 0.7

    Distribution: Gamma, Number of SNPs: 20, h^2: 0.7

    Distribution: Gaussian, Number of SNPs: 50, h^2: 0.7

    Distribution: Gaussian, Number of SNPs: 150, h^2: 0.4

Each scenario is repeated 50 times, producing 50 different phenotypes and sets of associated SNPs, creating enough simulation instances for statistical evaluation.

2. **Sample Splitting.** For each repetition and scenario, we choose to hold out a percentage of 10% of the samples which will be used to test the final model produced by the automated pipeline. Furthermore, a 50% of the remaining samples will be used for training (feature selection and modelling), while the rest 50% will be used to estimate the performance of the training procedure. Each feature selection algorithm, *εpilogi* and *QTCAT* will be trained, validated and finally tested on exactly the same sample splits.
3. **Modelers.** Although JADBIO is able to utilize numerous modelling algorithms, such as *Support Vector Machines, Random Forests* and *Ridge Regression*, for the simulation properties we will use only *Random Forests.* This choice stems from the fact that we are interested more in a relative comparison between the two feature selection methods and an exhaustive search of the universally best modelling method is not of grave importance here. For the same reason, regarding also computational time speedup, we limit the number of hyper-parameters of RFs to; $meanLeafSize = [5, 10]$, $numPredictorsToSample = [0.5, 1, 1.5]$ and $numTrees = 1000$, totaling to 6 modelling configurations.
4. **Selection Threshold.** Both feature selection methods incorporate a parameter which controls the number of features selected. *QTCAT* uses a p-value criterion, where higher values indicate larger signature size and less control over false positive features. On the other hand, *εpilogi* uses ΔBIC, the relative drop of Bayesian Information Criterion (BIC) scores of two successive statistical models. Here, larger values of BIC correspond to stricter selection criteria, thus p-value and BIC are reversely proportional. Generally, BIC form is given by:

$$BIC = -2ln(\hat{L}) + k \cdot ln(n)$$

where $\hat{L}$ the maximized value of the likelihood function of the model, while for regression tasks:

$$BIC = n \cdot ln(\frac{2\pi \sum e_i}{n}) + n + k \cdot ln(n)$$

where, n: number of samples, e_i: current model's residuals per sample, k: difference in number of independent variables in comparing models. Residuals cannot be known beforehand, so this type of BIC obstructs the determination of threshold parameters for *εpilogi*, however when sample size is sufficiently large BIC can be rewritten as:

$$BIC = X^2_{1-a,df} + k \cdot ln(n)$$

and for $df = 1$ the formula reduces to:

$$BIC = X^2_{1-a,df} + ln(n)$$

where, $X^2_{1-a,df}$ the statistic of a chi-square distribution with $1 - a$ confidence level and 1 degree of freedom. This formula depends only on sample size and a predefined significance level, allowing a direct comparison with *QTCAT*. For the purposes of these scenarios we used 10 p-values logarithmically spaced between the range 10^{-6} and 0.8, for *QTCAT* and the corresponding BIC values for *εpilogi*.

All scenarios in comparing predictive performance and feature selection of *εpilogi* and *QTCAT*

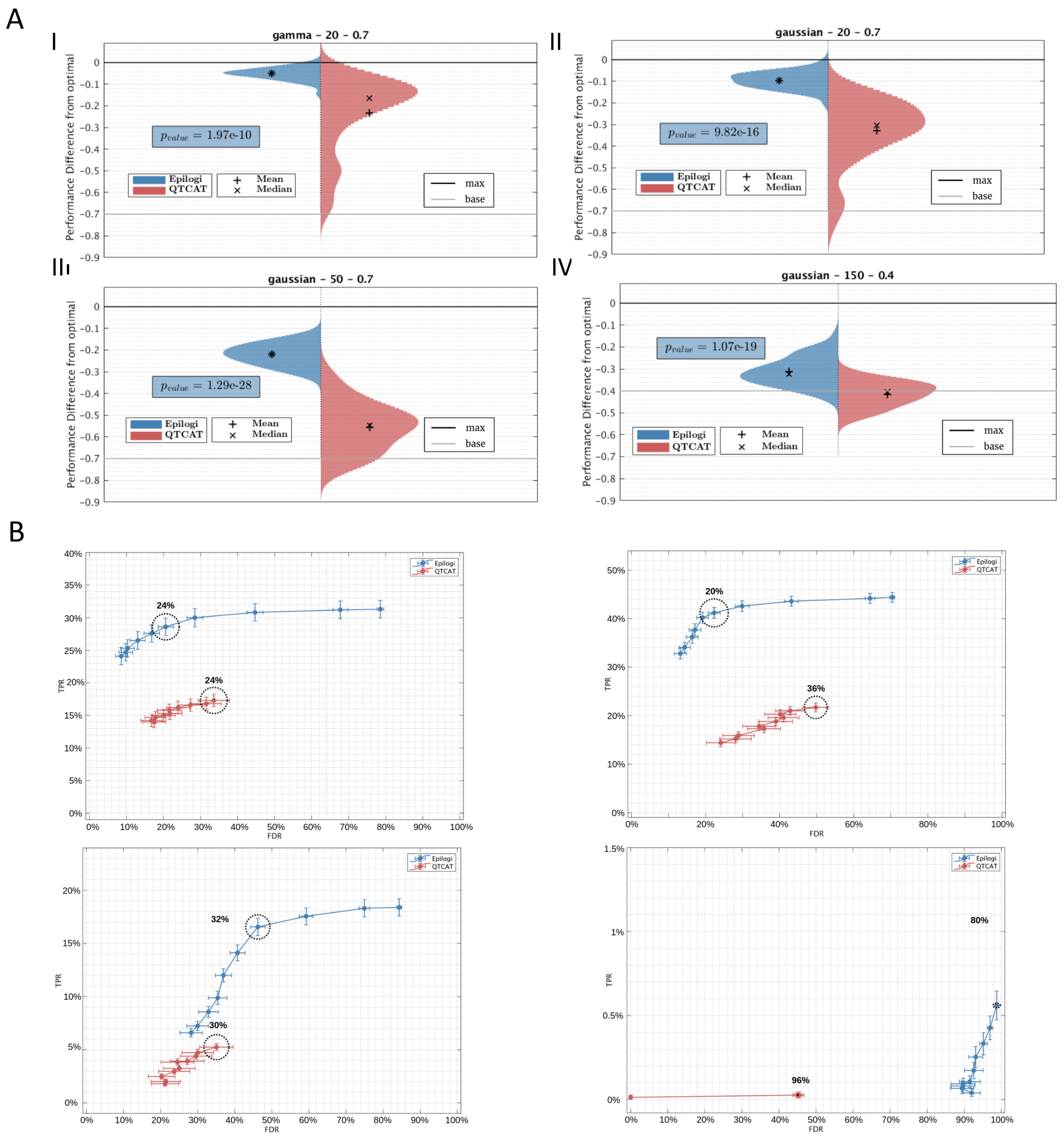

Supplementary figure 1: Distribution of differences of performances of the best models using signatures selected by εpilogi (light blue) and QTCAT (red) from the theoretical optimal model. The horizontal line base is the difference with the baseline model that always predicts the mean value of the outcome, and line max is the maximum difference from the optimal that can be achieved. The p-value of a t-test comparing the means of the distributions is shown. Epilogi discovers signatures that are statistically significantly more predictive than QTCAT. (B) Average True Positive Rate (TPR) and False Discovery Rate (FDR) of causal variants identification across 10 p-value thresholds for QTCAT and εpilogi. The threshold most frequently selected by JADBio when optimizing model performance is circled in dotted line, while the percentage of selection lies right above. Epilogi dominates QTCAT in both TRP and FDR. The threshold that most frequently optimizes performance achieves a balance between TRP and FDR, which is not true for QTCAT, while circle radius is inversely proportional to this frequency.

Genomic views of the variants and genes associated with diseases and their impact on protein function

Ankylosing Spondylitis

JADBio with γ-OMP

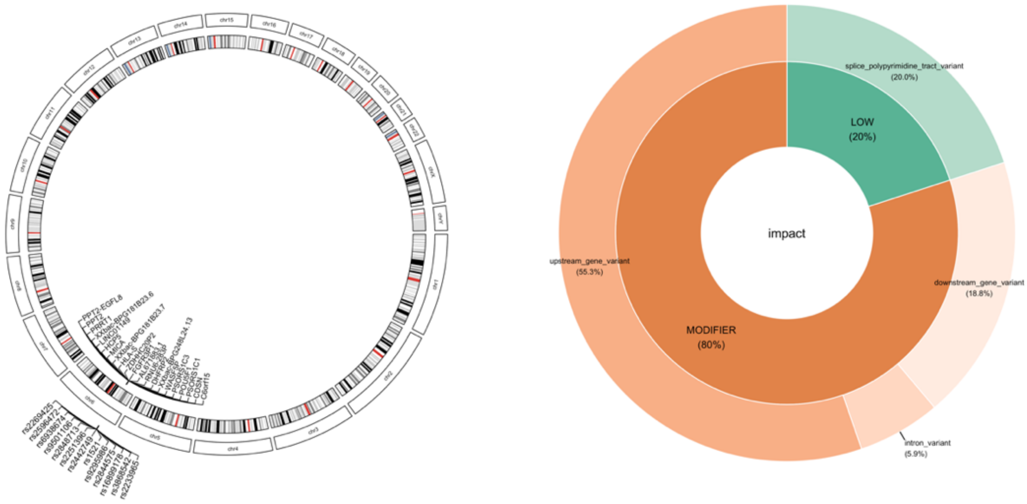

Published Study

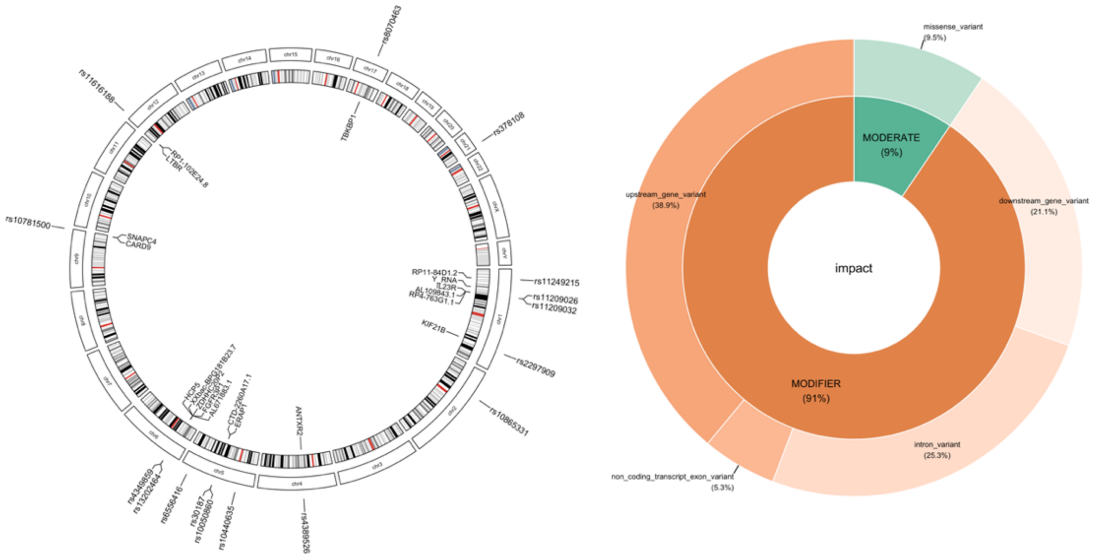

Supplementary Figure 2: Left: Genomic view of the variants and genes associated with Ankylosing Spondylitis (top: variants and genes discovered by JADBio-Gεn, bottom: variants and genes discovered by the original study).

Parkinson’s

JADBio with γ-OMP

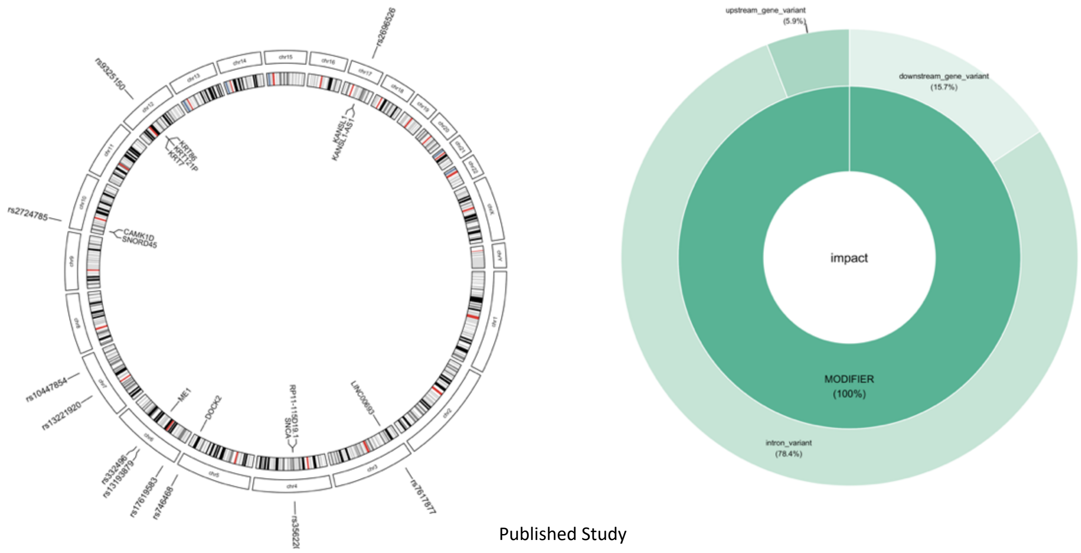

Published Study

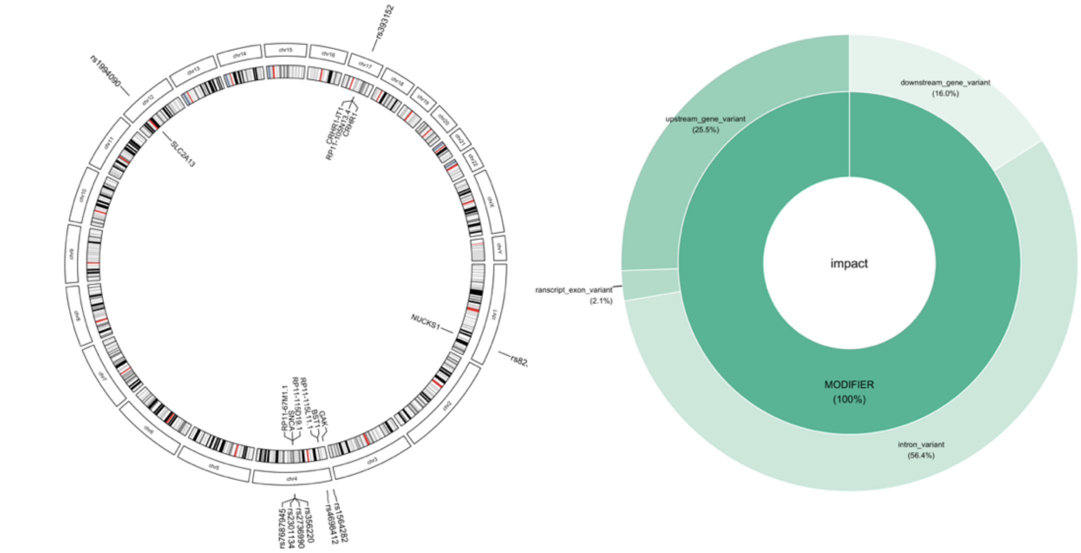

Supplementary Figure 3: Left: Genomic view of the variants and genes associated with Parkinson’s disease. (top: variants and genes discovered by JADBio-Gєn, bottom: variants and genes discovered by the original study).

Psoriasis

JADBio with γ-OMP

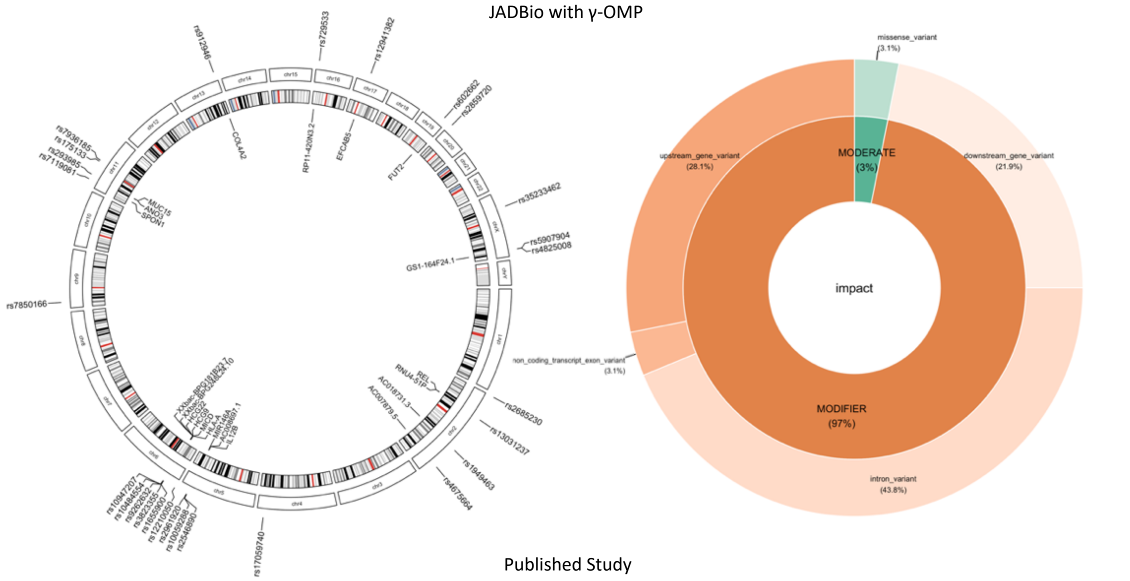

Published Study

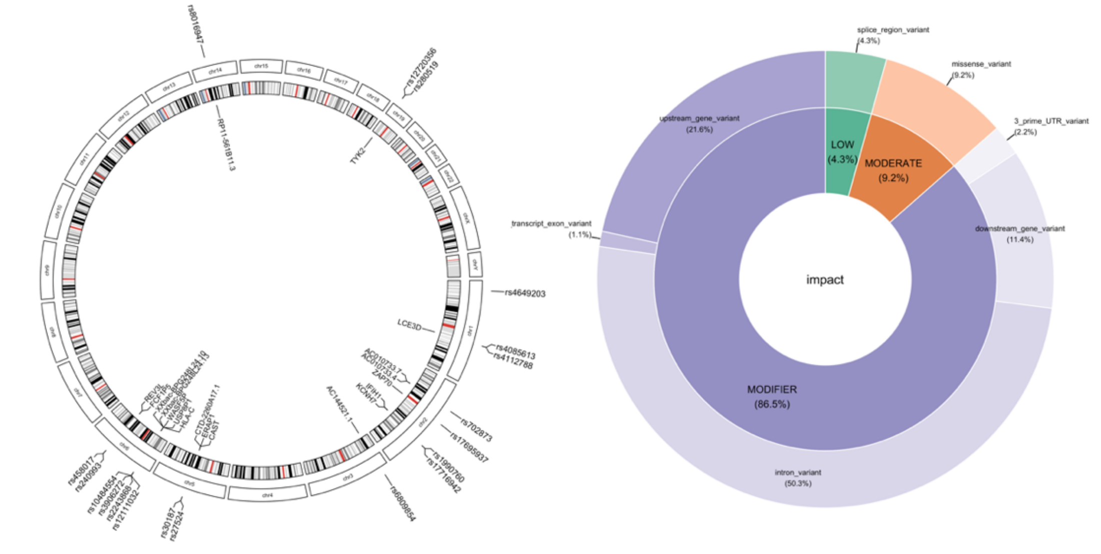

Supplementary Figure 4: Left: Genomic view of the variants and genes associated with Psoriasis (top: variants and genes discovered by JADBio-Gεn, bottom: variants and genes discovered by the original study).
